# Gold and silver as safe havens: A fractional integration and cointegration analysis

**DOI:** 10.1371/journal.pone.0282631

**Published:** 2023-03-03

**Authors:** Guglielmo Maria Caporale, Luis Alberiko Gil-Alana

**Affiliations:** 1 Brunel University London, London, United Kingdom; 2 University of Navarra, Pamplona, Spain; 3 Universidad Francisco de Vitoria, Madrid, Spain; University of Durham: Durham University, UNITED KINGDOM

## Abstract

This paper investigates whether gold and silver can be considered safe havens by examining their long-run linkages with 13 stock price indices. More specifically, the stochastic properties of the differential between gold/silver prices and 13 stock indices are analysed applying fractional integration/cointegration methods to daily data, first for a sample from January 2010 until December 2019, then for one from January 2020 until June 2022 which includes the Covid-19 pandemic. The results can be summarised as follows. In the case of the pre-Covid-19 sample ending in December 2019, mean reversion is found for the gold price differential only vis-à-vis a single stock index (SP500). whilst in seven other cases, although the estimated value of d is below 1, the value 1 is inside the confidence interval and thus the unit root null hypothesis cannot be rejected. In the remaining cases the estimated values of d are significantly higher than 1. As for the silver differential, the upper bound is 1 only in two cases, whilst in the others mean reversion does not occur. Thus, the evidence is mixed on whether these precious metals can be seen as safe havens, though it appears that this property characterises gold in a slightly higher number of cases. By contrast, when using the sample starting in January 2020, the evidence in favour of gold and silver as possible safe havens is pretty conclusive since mean reversion is only found in a single case, namely that of the gold differential vis-à-vis the New Zealand stock index.

## 1. Introduction

This paper investigates whether gold and silver can be considered safe havens by examining their long-run relationship with 13 stock price indices. For our purposes, assets are defined as safe havens if they are not linked in the long run to stock prices and thus protect investors’ wealth from movements in financial markets over long time horizons. This is a more general definition than others previously adopted in the literature which focused instead on crisis periods only and distinguished between weak and strong safe havens requiring no or negative correlation with stock prices respectively during episodes of financial turmoil; moreover, a perfect negative correlation is said to characterise a hedge since in such cases a portfolio including both types of assets will have a zero variance around the mean return [1].

A number of studies focus on the short-run links between gold and financial assets and report mixed results. For instance [2], argued that gold is an effective hedge, whilst [3] concluded that this is the case only intermittently, and [4] also found an episodic role as a hedge but only against inflation [5]. Provided evidence that in the US, UK and Germany during times of financial turbulence gold is a hedge for stocks (i.e. it is negatively correlated) and it is also a safe haven in the short run (i.e. the sum of the coefficients on stocks and some interactive variables is negative or zero) [6]. Considered a wider set of countries and various data frequencies and obtained different results depending on the countries and periods examined [1]. Used forward instead of spot gold prices and tested for cointegration with stock returns in the long run and during crisis periods as well as analysing their conditional covariance; in brief, their results confirm those of [5], since they imply that gold is a strong safe haven only in the short run and only in some countries and crisis periods [7]. Estimated a smooth transition regression (STR) using an exponential transition function and considering two different regimes corresponding to normal and extreme market conditions respectively, the latter being characterised by high volatility of stock returns, to establish whether gold can be regarded as a hedge or a safe heaven. Their results, based on 18 individual markets as well as five regional indices for the period running from January 1970 to March 2012 at a monthly frequency, indicate that gold can play both roles, but there are differences across countries.

Other papers examine the inflation hedge effectiveness of gold using cointegration techniques, in most cases estimating a standard vector error correction model (VECM) and obtaining mixed results–see, e.g., [7–11]. More recently [12], showed that a Markov-switching VECM is more appropriate in this context and concluded that gold is able to hedge future inflation in the long run only to some extent and more in the US and Japan than in the UK and the Euro Area.

As for silver [13, 14], Batten et al. (2010, 2014) showed that different precious metals have different features such that they cannot be considered as a single asset; therefore [15] extended the analysis to examine the safe haven properties of four precious metals (gold, silver, platinum and palladium) by estimating a Dynamic Conditional Correlation (DCC) model, and found that there are periods when silver, platinum and palladium act as safe havens whilst gold does not, and when they all do silver is a more effective safe haven than gold against stock price falls.

A few recent studies have focused specifically on the Covid-19 period. For instance [16], used a multivariate asymmetric dynamic conditional correlation (DCC) model and found that during the pandemic gold was a weak safe haven, while Bitcoin was not effective in that respect owing to its higher variability [17]. Applied a DCC-GARCH framework to high-frequency data on various stock indices distinguishing between different phases of the pandemic corresponding to different fiscal and monetary responses; they reported that gold served as a safe haven only in the early stages of the pandemic and became instead a ‘flight-to-safety’ asset in the later stages, during which hedging costs increased. Also [18], obtained evidence that cryptocurrencies were more effective than gold for hedging purposes during the Covid-19 crisis, whilst [19] found higher connectedness between gold price returns and cryptocurrency returns during the first wave of the pandemic.

Following the definition of safe havens specified above, the present paper focuses on the long-run relationship between gold and silver prices in turn and stock market prices (all in logs) by analysing the properties of their differential. Compared to earlier studies it provides thorough evidence for 13 stock markets based on a more general modelling framework. More specifically, it examines the stochastic properties of the differential between gold/silver prices and stock prices using fractional integration/cointegration methods. Unlike traditional methods based on the stationary/nonstationary I(0)/I(1) dichotomy our approach allows the differencing parameter to take any real values, including fractional ones, and thus it encompasses a much wider range of dynamic processes, including cases when mean reversion occurs but at a very low speed. The analysis is carried out first for a pre-Covid sample and then for the pandemic period with the aim of establishing whether the extent to which precious metals such as gold and silver can be used as safe havens differs between normal and crisis periods. Note that, whilst a few studies have used fractional integration methods to analyse persistence in gold and silver prices (see, e.g., [20–24], none have applied this method to examine their possible role as safe havens, which is instead our focus. The paper is organised as follows. Section 2 outlines the methodology. Section 3 describes the data and discusses the empirical results. Section 4 offers some concluding remarks.

## 2. Methodology

The empirical analysis is based on the concept of fractional integration, which allows the differencing parameter d to be any real value, including fractional ones. More precisely, assuming that {x_t_, t = 0, ±1, …} is an integrated of order 0 or I(0) process, defined as a covariance stationary process with a spectral density function which is positive and bounded at all frequencies, a process is said to be integrated of order d or I(d) if it can be expressed as:

$${\left(1-B\right)}^{d}x_{t}=u_{t},\;t=0,\pm 1,\ldots ,$$ (1)

where B is defined as the backshift operator implying that B^n^x_t_ = x_t-n_.

If d > 0, x_t_ displays the property of long memory and its spectral density function is unbounded at the zero frequency. Using a binomial expansion, the polynomial in B in (1) can be expressed as

$${\left(1-B\right)}^{d}=\sum_{j=0}^{\infty }\frac{\Gamma (j-d)}{\Gamma (j+1)\Gamma (-d)}B^{j},$$ (2)

where *Γ(x)* is the Gamma function, or alternatively as

$${\left(1-B\right)}^{d}=\sum_{j=0}^{\infty }(\begin{array}{l} d\\ j \end{array}){\left(-1\right)}^{j}B^{j}=1-dB+\frac{d(d-1)}{2}B^{2}-\ldots$$ (3)

and thus x_t_ can be expressed in terms of all its history.

In the empirical application discussed in the following section, x_t_ in (1) are the errors in a regression model that includes an intercept and a linear time trend, i.e.,

$$y_{t}=\alpha +\beta t+x_{t},\;t=1.2,\ldots ,$$ (4)

where y_t_ stands for the gold (silver) price-stock price differential (in logs) and α and β are unknown parameters to be estimated, namely the constant and the time trend coefficient. Note that Eqs (1) and (4) can be written together as:

$${\tilde{y}}_{t}=\alpha {\tilde{1}}_{t}+\beta {\tilde{t}}_{t}+u_{t},\;t=1.2,\ldots$$ (5)

where

$${\tilde{y}}_{t}={\left(1-B\right)}^{d}y_{t};\;{\tilde{1}}_{t}={\left(1-B\right)}^{d}1;\;{\tilde{t}}_{t}={\left(1-B\right)}^{d}t,$$ (6)

and u_t_ in (5) is I(0) by assumption, which implies that standard t-tests are valid. Following [25] the estimation is carried out using a Whittle function in the frequency domain as in many other long-memory studies.

Note that another possibility would be to test for cointegration between gold and silver prices respectively and each of the stock price indices considered following the two-step approach proposed in the seminal paper by [26]. In the first step one carries out unit root tests ([27–29], etc.) (or I(d) tests in the context of fractional integration) to establish if the individual series are I(1) (or I(d)). Note, however, that several studies show that standard unit root methods have very low power if the true data generating process (DGP) is fractionally integrated ([30–32], etc.). Then in the second step one checks if there exists a linear combination of each pair of variables which is stationary, i.e. whether the residuals x_t_ from the following equation are I(0) (or I(b) with b < d, namely whether the two series are fractionally cointegrated–see [33, 34]. Although in the original paper of [26] the orders of integration in the individual series and the cointegrating relationship (i.e., d and d-b respectively) were allowed to be fractional values, most of the empirical applications of this method only use integer values, i.e., 1 for the order of integration of the individual series and 0 for the cointegrating relationship, i.e., d = b = 1:

$$SAFEHAVEN_{t}=a+bSPM_{t}+y_{t},\;t=1.2,\ldots$$ (7)

where SAFE HAVEN_t_ stands for the log of gold and silver prices in turn, and SMP_t_ for the log of each of the stock indices considered. If the residuals are I(0) there is cointegration in the classical sense; if they are I(1) or I(d, d> 1) there is no cointegration, and, finally, if they are I(d, d < 1) mean reversion occurs but the dynamic adjustment towards the long-run equilibrium is slow.

The simpler approach adopted here is to assume that both SAFE HAVEN_t_ and SMP_t_ are I(1) and a = 0 and b = 1 (this is in fact confirmed by standard unit root tests; these results are not reported for reasons of space but are available upon request), in the above regression, i.e. to calculate the difference between the two variables and then test for the (fractional) order of integration of the corresponding residuals y_t_ as in [35]. A significant advantage of this strategy is that one can use the observed data (based on the differentials) rather than the estimated values for obtaining estimates of the fractional differencing parameters. Also note that estimating d from the errors in a regression model as in the [26] seminal paper would require the computation of critical values for that parameter. Therefore, testing mean reversion (i.e., d < 1) on the differential seems a convenient approach to follow in this context.

## 3. Data and empirical results

The dataset comprises gold and silver prices as well as 13 stock indices, more specifically daily closing values for two different subsamples. The first one goes from 4 January 2010 to 31 December 2019, whilst the second one goes from 2 January 2020 to 3 June 2022 and thus includes the Covid-19 pandemic. The indices considered are BFX (BEL20, Brussels), CAC40 (Paris), DOW (Dow Jones), HSI (Hang Seng Index), N100 (Euronext 100 Index), NAS (Nasdaq 100), NIK (Nikkei 225), NYA (NYSE Composite), NZX (NZX Limited), RUT (Russell 2000), SP5 (S&P 500), STO (Stockholm Stock Index) and XAX (NYSE AMEX Composite Index). The source is Yahoo Finance for all series. Standard methods have been used to calculate missing values. Specifically, we compute arithmetic means with respect to the previous and posterior values. This only occurred in a very small number of cases, namely less than 0.001% of all observations.

Concerning the sample selection, in order to obtain comparable results we have only included aggregate indices for developed countries. The fact that the composition of these indices varies does not affect the analysis since this is conducted on a pairwise basis: we examine whether gold and silver can be used as safe havens in the case of each individual market, and therefore differences between markets do not affect the validity of the results. Note that we have chosen to focus on developed markets only because these, given their size, provide the most significant investment opportunities–by comparison the developing ones only offer relatively small ones and therefore are of lesser interest.

We estimate the following regression model:

$$y_{t}=\alpha +\beta t+x_{t},\;{\left(1-B\right)}^{d}x_{t}=u_{t},\;t=1.2,\ldots$$ (8)

where u_t_ is I(0) or a short-memory process.

Tables 1–4 display the estimates of d along with the 95% confidence bands for the differencing parameter for three different specifications, namely i) no deterministic terms, i.e. α = β = 0 in (8); ii) only a constant, i.e., β = 0 in (8); and iii) a constant and a linear time trend. The coefficients in bold are those from the model selected in each case on the basis of the statistical significance of the regressors. It is assumed that the error term u_t_ in (8) is weakly autocorrelated. However, instead of imposing a standard ARMA model specification we follow the exponential spectral approach of [36] which is very suitable in the context of fractional integration.

**Table 1:** Estimates of d for the GOLD differential. Sample ending in Dec. 2020.

Series	No terms	An intercept	An intercept and a linear time trend
BFX	0.97 (0.91, 1.00)	**0.95 (0.91, 1.00)**	0.95 (0.91, 1.00)
CAC	0.97 (0.93, 1.03)	**0.95 (0.90, 1.00)**	0.95 (0.90, 1.00)
DOW	0.98 (0.94, 1.03)	0.95 (0.90, 1.00)	**0.94 (0.90, 1.00)**
HSI	0.99 (0.95, 1.04)	**0.99 (0.95, 1.05)**	0.99 (0.95, 1.05)
N100	0.98 (0.94, 1.03)	**0.95 (0.91, 1.00)**	0.95 (0.91, 1.00)
NAS	0.96 (0.92, 1.01)	0.95 (0.91, 1.00)	**0.95 (0.90, 1.00)**
NIK	0.99 (0.94, 1.04)	**0.99 (0.95, 1.05)**	0.99 (0.95, 1.05)
NYA	0.99 (0.94, 1.04)	**0.94 (0.90, 1.00)**	0.94 (0.90, 1.00)
NZX	0.97 (0.93, 1.02)	1.00 (0.96, 1.05)	**1.00 (0.96, 1.05)**
RUT	0.98 (0.93, 1.03)	**0.96 (0.92, 1.02)**	0.96 (0.92, 1.02)
SP5	0.94 (0.90, 0.99)	0.94 (0.89, 0.99)	**0.94 (0.89, 0.99)**
STO	0.97 (0.93, 1.02)	**0.96 (0.92, 1.01)**	0.96 (0.92, 1.01)
XAX	0.96 (0.92, 1.02)	**0.96 (0.91, 1.02)**	0.96 (0.91, 1.01)

Note: in bold, the selected model according to statistical significance of the deterministic terms; in red: evidence of mean reversion at the 95% level; in brackets, the 95% confidence intervals.

**Table 2:** Estimates of d for the SILVER differential. Sample ending in Dec. 2020.

Series	No terms	An intercept	An intercept and a linear time trend
BFX	1.00 (0.95, 1.05)	**0.96 (0.92, 1.01)**	0.96 (0.92, 1.01)
CAC	0.99 (0.95, 1.05)	**0.96 (0.91, 1.00)**	0.96 (0.91, 0.99)
DOW	1.00 (0.95, 1.05)	**0.96 (0.92, 1.02)**	0.96 (0.92, 1.02)
HSI	0.99 (0.95, 1.04)	**1.00 (0.95, 1.05)**	1.00 (0.95, 1.05)
N100	0.99 (0.95, 1.04)	**0.96 (0.92, 1.00)**	0.96 (0.92, 1.00)
NAS	0.99 (0.95, 1.04)	0.97 (0.93, 1.03)	**0.97 (0.93, 1.03)**
NIK	1.00 (0.96, 1.05)	**0.99 (0.95, 1.04)**	0.99 (0.95, 1.04)
NYA	1.00 (0.94, 1.04)	**0.97 (0.93, 1.01)**	0.97 (0.93, 1.01)
NZX	0.99 (0.95, 1.04)	**0.99 (0.95, 1.04)**	0.99 (0.95, 1.04)
RUT	0.99 (0.95, 1.04)	**0.97 (0.93, 1.02)**	0.98 (0.93, 1.02)
SP5	0.99 (0.95, 1.04)	**0.96 (0.92, 1.02)**	0.96 (0.92, 1.02)
STO	0.99 (0.95, 1.04)	**0.97 (0.93, 1.02)**	0.97 (0.93, 1.02)
XAX	1.00 (0.96, 1.03)	**0.96 (0.91, 1.02)**	0.96 (0.91, 1.02)

Note: in bold, the selected model according to statistical significance of the deterministic terms; in red: evidence of mean reversion at the 95% level; in brackets, the 95% confidence intervals.

**Table 3:** Estimates of d for the GOLD differential. Sample ending in June 2022.

Series (with respect to gold)	No terms	An intercept	An intercept and a linear time trend
**BFX**	0.97 (0.88, 1.08)	**0.99 (0.91, 1.12)**	0.99 (0.91, 1.13)
**CAC**	0.97 (0.88, 1.08)	**1.01 (0.93, 1.14)**	1.01 (0.93, 1.13)
**DOW**	1.00 (0.91, 1.10)	**1.00 (0.88, 1.12)**	1.00 (0.87, 1.12)
**HSI**	0.96 (0.87, 1.08)	**0.93 (0.85, 1.04)**	0.93 (0.86, 1.04)
**N100**	1.03 (0.95, 1.13)	**1.02 (0.94, 1.13)**	1.02 (0.94, 1.13)
**NAS**	0.95 (0.87, 1.07)	**0.92 (0.86, 1.03)**	0.92 (0.86, 1.03)
**NIK**	0.97 (0.88, 1.07)	**1.00 (0.91, 1.11)**	0.99 (0.91, 1.10)
**NYA**	0.96 (0.88, 1.08)	**0.97 (0.91, 1.10)**	0.97 (0.91, 1.10)
**NZX**	0.98 (0.90, 1.09)	**0.84 (0.72, 0.97)**	0.84 (0.74, 0.97)
**RUT**	1.02 (0.93, 1.14)	**1.04 (0.94, 1.15)**	1.04 (0.94, 1.15)
**SP5**	1.02 (0.95, 1.12)	**0.97 (0.86, 1.11)**	0.97 (0.87, 1.11)
**STO**	0.95 (0.86, 1.08)	**0.99 (0.90, 1.12)**	0.99 (0.91, 1.12)
**XAX**	1.02 (0.94, 1.14)	**1.10 (1.00, 1.23)**	1.10 (1.00, 1.23)

Note: in bold, the selected model according to statistical significance of the deterministic terms; in red: evidence of mean reversion at the 95% level; in brackets, the 95% confidence intervals.

**Table 4:** Estimates of d for the SILVER differential. Sample ending in June 2022.

Series (with respect to silver)	No terms	An intercept	An intercept and a linear time trend
**BFX**	0.99 (0.90, 1.08)	**1.09 (0.97, 1.24)**	1.09 (0.97, 1.24)
**CAC**	0.99 (0.90, 1.10)	**1.09 (0.98, 1.25)**	1.09 (0.98, 1.25)
**DOW**	0.99 (0.91, 1.10)	**1.00 (0.91, 1.11)**	1.00 (0.91, 1.11)
**HSI**	0.98 (0.89, 1.10)	**0.98 (0.88, 1.11)**	0.98 (0.88, 1.11)
**N100**	0.99 (0.90, 1.10)	**1.10 (0.99, 1.25)**	1.10 (0.99, 1.25)
**NAS**	0.98 (0.88, 1.09)	**1.05 (0.94, 1.20)**	1.05 (0.94, 1.20)
**NIK**	0.98 (0.89, 1.10)	**1.02 (0.90, 1.17)**	1.02 (0.90, 1.17)
**NYA**	0.97 (0.88, 1.10)	**1.07 (0.95, 1.21)**	1.07 (0.95, 1.21)
**NZX**	0.98 (0.89, 1.10)	**1.01 (0.92, 1.14)**	1.01 (0.92, 1.14)
**RUT**	0.97 (0.87, 1.08)	**1.06 (0.95, 1.20)**	1.06 (0.95, 1.20)
**SP5**	0.99 (0.91, 1.10)	**1.01 (0.92, 1.12)**	1.01 (0.92, 1.12)
**STO**	0.98 (0.90, 1.10)	**1.08 (0.96, 1.22)**	1.08 (0.96, 1.22)
**XAX**	0.99 (0.88, 1.08)	**1.13 (1.02, 1.28)**	1.13 (1.02, 1.28)

Note: in bold, the selected model according to statistical significance of the deterministic terms; in red: evidence of mean reversion at the 95% level; in brackets, the 95% confidence intervals.

It can be seen from Table 1 that for the gold differentials the time trend is significant only in four cases (vis-à-vis DOW, NAS, NZ50, and SP5). Evidence of mean reversion is found only in a single case (vis-à-vis SP5), whilst in seven other cases (vis-à-vis BFX, CAC, DOW, N100, NAS, and NYA), although the estimated value of d is below 1, the value 1 is inside the confidence interval and thus the unit root null hypothesis cannot be rejected. In the remaining cases the estimated values of d are significantly higher than 1. The corresponding results for the silver price differential are reported in Table 2. In this case the time trend is significant for the differential vis-à-vis NAS, and although there are two cases which are close to mean reversion (vis-à-vis CAC and N100) the value of 1 is in the upper bound region of the interval, and thus the hypothesis of mean reversion is close to being rejected. Note that our sample includes various indices for the US, some of a more general nature (NYA and SP5), some based on large cap stocks only (DOW, NAS), and one for small cap stocks only (RUT). Our results suggest that there are no differences between small and large cap stocks in terms of the possible role of gold and silver as safe havens, since in neither case is mean reversion observed, whilst it is found in the case of the wider indices.

Next we investigate whether the relationships of interest were different during the Covid-19 pandemic by redoing the estimation over the period from January 2020 to June 2022. These results are reported in Tables 3 and 4 for the differentials with respect to gold and silver respectively. In contrast to the previous period, mean reversion is not found in any case for the silver differentials whilst it only occurs vis-à-vis NZX in the case of gold; in all other cases the estimates of d are equal to or higher than 1. It is clear therefore that during the pandemic both precious metals considered could very effectively be used as a safe haven. Again, no differences are found between small and large cap stocks in this respect.

## 4. Conclusions

This paper analyses the stochastic properties of the differential between gold and silver prices in turn and 13 stock price indices using fractional integration methods. The aim is to establish whether gold and silver can be considered safe havens in the sense that there exist no long-run linkages with stock prices and thus these assets are insulated from stock market developments; the analysis is carried out for both a pre-Covid sample and for the pandemic period to establish whether gold and silver can be seen as safe havens in either normal or crisis periods. The wide country coverage combined with the focus on the long run and the more general modelling approach allowing for a variety of cases including slow mean reversion differentiate the present study from previous ones.

The results can be summarised as follows. When considering the pre-Covid sample, under the assumption of weakly autocorrelated disturbances mean reversion is only found for the gold price differential vis-à-vis SP5, and for another group of seven indices (BFX, BSE, CAC, DOW, N100, NAS, and NYA) the value 1 for the differencing parameter is in the upper region of the confidence interval. In the case of the silver differentials vis-à-vis CAC and N100 the value 1 is also in the upper region of the confidence interval. Therefore the evidence is mixed on whether these precious metals can be seen as safe havens, though it appears that this property characterises gold in a slightly higher number of cases. These results are consistent with the ones previously obtained by other researchers such as [1, 5, 15], who also reported mixed evidence, though in our case this concerns more specifically the long-run equilibrium allowing for the possibility of a very slow dynamic adjustment towards it. However, the results for the Covid-19 period are pretty conclusively supporting the possibility of using gold and silver as safe havens, since mean reversion occurs only for a single gold differential and for none of the silver ones. This is broadly consistent with the evidence in favour of gold as a weak safe heaven reported by [16], but in contrast to the findings by [17] according to which gold served as one only in the first phase of the pandemic; however, it should be noted that both these studies use a DCC framework rather than long-memory methods focusing on the long run as in our case.

The implication of our results is that investing in precious metals is not equally appealing in normal vis-à-vis crisis periods. During the former, it remains a moot question whether gold and silver can be used effectively as safe havens: appropriate investment strategies should be designed in each case taking into account the properties of individual markets whilst general investment rules clearly do not apply. During the latter periods, it appears that investors can use gold and silver to protect their portfolio from the effects of negative shocks to stock markets, which do not get transmitted to precious metals. Future work should analyse whether gold and silver at such times are also insulated from developments in other types of financial markets given some evidence suggesting that cryptocurrencies rather than gold had the potential to control risk during the Covid-19 crisis (see [18]) and that connectedness between gold price returns and cryptocurrency returns increased sharply during the first wave of the pandemic (see [19]).
